# The Impact of the COVID-19 Pandemic on Surgical Services in Brazil's Healthcare System: A Retrospective Cross-Sectional Study

**DOI:** 10.7759/cureus.44693

**Published:** 2023-09-04

**Authors:** Billy McBenedict, Wilhelmina N Hauwanga, Dulci Petrus, God-dowell O Odukudu, Gabriel de Moraes Mangas, Maria I do Nascimento

**Affiliations:** 1 Department of General and Specialized Surgery, Faculty of Medicine, Universidade Federal Fluminense, Niteroi, BRA; 2 Department of General and Specialized Surgery, Faculty of Medicine, Universidade Federal do Estado do Rio de Janeiro, Rio de Janeiro, BRA; 3 Department of Family Health, Ministry of Health and Social Services, Windhoek, NAM; 4 Department of Internal Medicine, Delta State University, Abraka, NGA

**Keywords:** surgery, pre-pandemic, post-pandemic, pandemic, length of stay, hospital admissions, emergency surgery, elective surgery, covid-19

## Abstract

Background

The coronavirus disease 2019 (COVID-19) pandemic provoked disruptions in healthcare delivery, leading to the cancellation and postponement of various health services, including surgery. Numerous countries closed their borders and established laws mandating the use of face masks and social distancing and enforced lockdowns, and various activities were constrained. Brazil, the largest and most populous country in Latin America, also experienced a rapid and sustained surge in infections and deaths. Brazil was the most severely impacted nation in Latin America. The impact of the pandemic on surgical services in Brazil has not been adequately studied since most studies only cover the early phases of the pandemic. Thus, this study aimed to assess the impact of the COVID-19 pandemic on surgical services throughout the entire period.

Methods

A retrospective cross-sectional design was used to examine surgical cases from 2019 to 2022 and compared the following indicators: (1) number of hospital admissions, (2) length of hospital stay (LOS) (in days), and (3) volume of urgent and elective procedures. Data was divided into four time periods, pre-pandemic (March-December 2019), pandemic (March-December 2020), recovery (March-December 2021), and post-pandemic (March-December 2022), and was analyzed for the number of admissions and LOS based on surgical procedures performed by stratifying according to region, sex, age, and type of surgery (urgent versus elective).

Results

The number of admissions for surgical procedures ranged between 859,646 and 4,015,624 for 2019, 686,616 and 3,419,234 for 2020, 787,791 and 3,829,019 for 2021, and 760,512 and 3,857,817 for 2022 for the category of region; 4,260,900 and 5,991,775 for 2019, 3,594,117 and 4,984,710 for 2020, 4,182,640 and 5,590,808 for 2021, and 4,077,651 and 5,561,928 for 2022 for the category of sex; and 2,170,288 and 3,186,117 for 2019, 1,516,830 and 2,825,189 for 2020, 1,748,202 and 3,030,272 for 2021, and 1,900,023 and 2,859,179 for 2022 for the category of age. The variable age showed a comparable trend, albeit with an expressive decline for surgeries in the age range of 0-19 years. The LOS (in days) for surgical procedures ranged between 110,157 and 910,846 for 2019, 58,562 and 897,734 for 2020, 67,926 and 904,137 for 2021, and 100,467 and 823,545 for 2022. Thoracic surgery indicated no statistically significant difference in the number of admissions and LOS. Elective surgeries had a decline in the number of admissions and LOS, a 13% and 9.3% decline between 2019 and 2020, respectively. Urgent surgeries experienced a slight decrease in admissions and LOS, with a decline of 2.4% and 2.8% between 2019 and 2020, respectively.

Conclusions

Population characteristics, such as age, sex, and region, showed decreased hospital admissions during the pandemic, followed by a recovery toward pre-pandemic levels afterward. The number of surgical admissions and the length of hospital stays decreased during the pandemic but gradually returned to pre-pandemic levels in the recovery and post-pandemic phases. Notably, thoracic surgery remained statistically consistent across all periods, indicating its emergency nature compared to other surgeries. Thus, we conclude that the pandemic had minimal impact on thoracic surgery cases, contributing to a stable trend.

## Introduction

In 2020, the World Health Organization (WHO) issued a global health alert regarding the emergence of a novel coronavirus, severe acute respiratory syndrome coronavirus 2 (SARS-CoV-2), in Wuhan, Hubei Province, China [1]. Succeeding its identification, a surge of coronavirus disease 2019 (COVID-19) cases was reported in several Asian countries, before spreading globally [2] and being declared a pandemic in March 2020. COVID-19 is highly contagious, and over 45 million infections in 219 countries were recorded by November 2021 [3]. COVID-19 has a lower lethality rate compared to other coronaviruses such as severe acute respiratory syndrome coronavirus (SARS-CoV) and Middle East respiratory syndrome coronavirus (MERS‐CoV) [4] but recorded a higher number of deaths owing to its significant transmissibility. SARS-CoV-2 is an enveloped positive-stranded RNA virus that primarily affects the respiratory system and can lead to multisystemic illness [5]. The typical clinical features of COVID-19 include fever, dry cough, dyspnea, anosmia, decreased oxygen saturation, elevated C-reactive protein levels, and pulmonary infiltrates [6]. The management of COVID-19 primarily relies on supportive care, in addition to vaccines that have shown promise in reducing the number of cases and hospitalizations in many countries [7].

The COVID-19 pandemic provoked disruptions in healthcare delivery, leading to the postponement of numerous elective surgical cases. As a result, some medical centers prioritized oncological and emergency surgical care [8]. Numerous countries closed their borders and established laws mandating the use of face masks and social distancing and enforced lockdowns. Social and business activities were constrained. It caused a strain on healthcare systems and delivery worldwide; for example, the USA experienced overcrowding of emergency rooms and a shortage of ICU facilities [9].

Brazil, the largest and most populous country in Latin America, is marked by pronounced socioeconomic inequality. Since reporting its first COVID-19 case on February 25, 2020, Brazil experienced a rapid and sustained surge in infections and deaths. As of March 16, 2023, the country recorded 37,085,520 confirmed cases and 699,310 deaths, making it the most severely impacted nation in Latin America, with São Paulo being the most affected region [7]. Brazil is divided into five regions and 27 federative units (UF), comprising 26 states and the capital city Brasília, as well as 5,570 municipalities. These geographical regions exhibit notable disparities in gross domestic product (GDP) and Human Development Index (HDI) [10,11].

Brazil's health system, Sistema Único de Saúde (SUS), was established in 1988 with principles of universality, comprehensiveness, equity, decentralization, and social participation [10]. SUS provides health coverage to approximately 80% of the nation's population [12]. Following the first recorded COVID-19 death, the Brazilian Ministry of Health recommended delaying or canceling elective surgeries regardless of virus prevalence and case distribution [13]. Most states had over a 50% reduction in low- to medium-complexity surgeries, transplants, and screenings in 2020 [10]. Non-COVID-19-related procedures also declined in 2020 due to hospital avoidance and appointment cancellation [14].

This study aimed to assess the impact of the COVID-19 pandemic on surgical services in Brazil's healthcare systems using a retrospective cross-sectional design. The researchers examined surgical cases from 2019 to 2022 and compared the following indicators during this period: (1) number of hospital admissions, (2) length of hospital stay (LOS), and (3) volume of urgent and elective procedures.

## Materials and methods

Study design

This study used a retrospective cross-sectional study design. A total of 16 subgroup surgical procedures from DATASUS were considered for analysis in this study. The data used was at the national level, and analysis was made at national and regional levels. The data was divided into four categories denoting four time periods in order to allow for better resolution in comparison. The four periods were pre-pandemic (March-December 2019), pandemic (March-December 2020), recovery (March-December 2021), and post-pandemic (March-December 2022). Data from the same months (March-December) for each year was deliberately used for consistency and ease of comparison. The pre-pandemic data was used as the baseline data for analysis in this study.

Data collection

Data was collected from the Brazilian Hospital Information System records under the "Departamento de Informática do SUS (DATASUS)," which is housed by the Sistema Único de Saúde (SUS), a Brazilian Unified Health System. DATASUS encompasses data from all hospitalizations reimbursed by SUS. It stands as a testament to Brazil's successful experience in the domain of health information management, a project spearheaded by the Federal Government of Brazil. DATASUS accumulates information from both public and private institutions affiliated with SUS.

Data analysis

The number of admissions for surgical procedures in the four periods stratified according to region, sex, and age range were presented using summary statistics (percentages). The data used for analysis was based on the total values, that is, the total number of admissions and the total number of days admitted (LOS). Data was analyzed for the number of admissions and LOS based on subgroups of surgical procedures performed during the pre-pandemic, pandemic, recovery, and post-pandemic periods. The number of admissions and LOS was also analyzed in relation to the type of surgery (urgent versus elective) performed during the same period. Data analysis was performed using Statistical Package for the Social Sciences (SPSS) version 27 (IBM SPSS Statistics, Armonk, NY, USA) as outlined in the literature [15]. A normality test was done using the Kolmogorov-Smirnov test, and non-normally distributed data was analyzed using the Kruskal-Wallis test to assess whether the differences were statistically significant across the four periods. For the same reason, a one-way analysis of variance (ANOVA) test was used for data that was normally distributed.

Ethical considerations

This study was conducted using deidentified secondary data, accessible through the DATASUS, and in compliance with Resolution 466/2012 of the National Health Council. This study did not require approval by the Research Ethics Committee.

## Results

Number of admissions based on population characteristics

For all regions, the number of admissions decreased during the pandemic period compared to baseline and thereafter generally increased close to baseline values (Table 1). A similar trend was observed with respect to sex: the number of admissions for both males and females considerably reduced in the pandemic period compared to baseline, followed by an increase. The age range showed a comparable trend in the number of admissions during the pandemic period, albeit with an expressive decline for the age range of 0-19 years, marked by a drop from 29.6% in 2019 to 20.7% in 2020. The analysis included the number of admissions for surgical procedures in the four periods, stratified according to region, sex, and age range (Table 1).

**Table 1:** Number of admissions for surgical procedures in the four periods stratified according to region, sex, and age range

	Pre-pandemic (March-December 2019)	Pandemic (March-December 2020)	Recovery (March-December 2021)	Post-pandemic (March-December 2022)
	Number (%)	Number (%)	Number (%)	Number (%)
Region				
North	859,646 (26.1)	748,236 (22.7)	869,646 (26.4)	814,308 (24.7)
North East	2,753,716 (27)	2,240,132 (22)	2,629,393 (25.8)	2,565,197 (25.2)
Southeast	4,015,624 (26.6)	3,419,234 (22.6)	3,829,019 (25.3)	3,857,817 (25.5)
South	1,823,357 (27.5)	1,484,609 (22.4)	1,657,603 (25)	1,665,088 (25.1)
Midwest	800,332 (26.4)	686,616 (22.6)	787,791 (26)	760,512 (25.1)
Sex				
Male	4,260,900 (26.4)	3,594,117 (22.3)	4,182,640 (26)	4,077,651 (25.3)
Female	5,991,775 (27.1)	4,984,710 (22.5)	5,590,808 (25.3)	5,561,928 (25.1)
Age range				
0-19 years	2,170,288 (29.6)	1,516,830 (20.7)	1,748,202 (23.8)	1,900,023 (25.9)
20-39 years	3,186,117 (26.8)	2,825,189 (23.7)	3,030,272 (25.5)	2,859,179 (24)
40-59 years	2,169,481 (25.9)	1,850,163 (22)	2,249,967 (26.8)	2,122,483 (25.3)
≥60 years	2,726,789 (25.7)	2,386,645 (22.5)	2,745,005 (25.9)	2,757,894 (26)

Number of admissions based on subgroups of procedures

The data pertaining to thoracic surgery indicated no statistically significant difference in the number of admissions across the mentioned periods (Table 2). However, for other types of surgical procedures, a statistically significant difference was observed. In contrast to thoracic surgery, these other types of surgical procedures exhibited higher admission rates during the pre-pandemic period, followed by a considerable decrease during the pandemic phase, and, subsequently, a gradual return toward the baseline during the recovery and post-pandemic periods. The results of the analysis included the number of admissions for surgical procedure subgroups in the four periods (Table 2).

**Table 2:** Number of admissions based on subgroups of surgical procedures performed during the study period

Procedure subgroup	Pre-pandemic (March-December 2019)	Pandemic (March-December 2020)	Recovery (March-December 2021)	Post-pandemic (March-December 2022)	P-value
	Number (%)	Number (%)	Number (%)	Number (%)	
Surgeries of the skin, subcutaneous tissue, and mucosa	110,157 (32.7)	58,562 (17.4)	67,926 (20.1)	100,467 (29.8)	0.000
Endocrine gland surgery	10,448 (34.6)	4,446 (14.7)	6,075 (20.1)	9,230 (30.6)	0.001
Central and peripheral nervous system surgery	77,927 (28.5)	57,739 (21.1)	65,919 (24.1)	72,260 (26.4)	0.001
Upper airway, face, head, and neck surgery	116,572 (32.8)	62,763 (17.7)	78,184 (22)	97,959 (27.6)	0.000
Surgery of the visual apparatus	102,365 (29.9)	56,018 (16.4)	78,310 (22.9)	105,613 (30.9)	0.000
Circulatory system surgery	253,391 (28.9)	185,836 (21.2)	207,296 (23.7)	228,933 (26.2)	0.000
Surgery of the digestive system, associated organs, and abdominal wall	692,630 (30.1)	412,913 (17.9)	493,159 (21.4)	704,860 (30.6)	0.000
Surgery of the musculoskeletal system	672,601 (26.5)	581,780 (22.9)	634,345 (25)	648,327 (25.6)	0.000
Surgery of the genitourinary system	458,050 (29.6)	258,155 (16.7)	330,542 (21.4)	500,185 (32.3)	0.000
Breast surgery	30,160 (34.2)	15,124 (17.1)	18,660 (21.1)	24,298 (27.5)	0.000
Obstetric surgery	910,846 (25.8)	897,734 (25.4)	904,137 (25.6)	823,545 (23.3)	0.001
Thoracic surgery	51,199 (25.4)	45,950 (22.8)	52,890 (26.3)	51,434 (25.5)	0.175
Reconstructive surgery	51,661 (30.4)	37,793 (22.2)	39,977 (23.5)	40,543 (23.9)	0.000
Oral and maxillofacial surgery	13,055 (31.7)	5,937 (14.4)	8,977 (21.8)	13,257 (32.2)	0.001
Surgery in oncology	134,743 (26.9)	112,445 (22.4)	124,770 (24.9)	128,973 (25.7)	0.042
Transplantation of organs, tissues, and cells	12,727 (30.8)	7,936 (19.2)	10,350 (25)	10,369 (25.1)	0.001

Length of hospital stay (days) according to subgroups of procedures

For thoracic surgery, the data indicated that there was no significant difference in the LOS during the four periods (Table 3). However, a significant difference in the LOS was observed with regard to other types of surgical procedures. With the exception of thoracic surgery, the percentages show an overall pattern of a longer LOS during the pre-pandemic, followed by the post-pandemic, and then the recovery period. The shortest LOS was observed during the pandemic period. The results of the analysis included the LOS for surgical procedure subgroups in the four periods (Table 3).

**Table 3:** LOS according to subgroups of surgical procedures performed during the study period LOS: length of hospital stay

Procedure subgroup	Pre-pandemic (March-December 2019)	Pandemic (March-December 2020)	Recovery (March-December 2021)	Post-pandemic (March-December 2022)	P-value
	Number (%)	Number (%)	Number (%)	Number (%)	
Surgeries of the skin, subcutaneous tissue, and mucosa	152,406 (31.8)	98,182 (20.5)	109,445 (22.8)	119,518 (24.9)	0.000
Endocrine gland surgery	27,815 (35.1)	12,223 (15.4)	16,030 (20.3)	23,068 (29.1)	0.000
Central and peripheral nervous system surgery	537,558 (27.7)	470,592 (24.2)	490,955 (25.3)	442,777 (22.8)	0.007
Upper airway, face, head, and neck surgery	327,829 (29.4)	245,474 (22)	286,252 (25.7)	254,787 (22.9)	0.000
Surgery of the visual apparatus	38,263 (35.4)	20,713 (19.2)	23,142 (21.4)	25,886 (24)	0.000
Circulatory system surgery	1,128,324 (29)	849,753 (21.8)	942,638 (24.2)	974,990 (25)	0.000
Surgery of the digestive system, associated organs, and abdominal wall	2,185,174 (29.4)	1,514,204 (20.4)	1,730,931 (23.3)	1,991,099 (26.8)	0.000
Surgery of the musculoskeletal system	2,920,282 (27.9)	2,351,044 (22.5)	2,587,166 (24.8)	2,594,280 (24.8)	0.001
Surgery of the genitourinary system	915,722 (30.3)	548,204 (18.1)	673,241 (22.3)	884,203 (29.3)	0.000
Breast surgery	48,946 (34.7)	26,189 (18.6)	29,905 (21.2)	36,121 (25.6)	0.000
Obstetric surgery	2,456,053 (26.8)	2,275,039 (24.8)	2,317,846 (25.3)	2,129,021 (23.2)	0.003
Thoracic surgery	458,478 (26.9)	371,699 (21.8)	427,419 (25.1)	445,659 (26.2)	0.075
Reconstructive surgery	241,722 (29)	196,946 (23.6)	204,019 (24.5)	191,175 (22.9)	0.000
Oral and maxillofacial surgery	12,688 (27.2)	8,280 (17.7)	11,729 (25.1)	13,997 (30)	0.003
Surgery in oncology	446,155 (28.5)	365,810 (23.4)	385,512 (24.6)	368,370 (23.5)	0.022
Transplantation of organs, tissues, and cells	118,621 (29)	88,737 (21.7)	103,144 (25.2)	98,057 (24)	0.001

Number of admissions and LOS (days) in relation to the type of surgery

There was a significant difference in the number of elective and urgent surgeries in relation to the number of admissions and LOS (Table 4). Elective surgeries had a pronounced decline in the number of admissions and LOS, a 13% and 9.3% decline between 2019 and 2020, respectively. This was followed by a return to baseline in the case of the number of admissions and a gradual increase toward baseline for LOS. Urgent surgeries experienced a slight decrease in admissions and LOS, with a decline of 2.4% and 2.8% between 2019 and 2020, respectively. However, subsequent to this decline, there was a gradual recovery, bringing both the number of admissions and the LOS back toward their baseline levels. The analysis included the number of admissions and LOS for surgical procedures in the four periods, stratified according to the type of surgery (Table 4).

**Table 4:** Number of admissions and LOS for elective and urgent surgeries performed during the four periods LOS: length of hospital stay

	Pre-pandemic (March-December 2019)	Pandemic (March-December 2020)	Recovery (March-December 2021)	Post-pandemic (March-December 2022)	P-value
Number of admissions					
Elective	2,235,284 (30.4)	1,283,692 (17.4)	1,609,928 (21.9)	2,228,403 (30.3)	0.001
Urgent	8,045,511 (26)	7,301,929 (23.6)	8,170,462 (26.4)	7,463,556 (24.1)	0.001
Length of stay					
Elective	7,922,509 (29.7)	5,442,183 (20.4)	6,444,618 (24.2)	6,832,280 (25.6)	0.001
Urgent	43,995,732 (26)	39,341,432 (23.2)	46,234,959 (27.2)	40,236,333 (23.7)	0.001

## Discussion

This study analyzed the number of admissions and LOS (days) associated with the different types of surgeries and surgical procedure subgroups performed.

Population characteristics

The population characteristics explored had a similar trend with a decline in the number of admissions during the pandemic, followed by an increase toward baseline during the recovery and post-pandemic period. However, the age range of 0-19 years had a notable decline (8.9%) in the number of admissions during the pandemic period (Table 1). This is possibly because, in addition to the decline in admissions across all age groups based on the lockdown, the adolescent group perhaps benefited from this measure due to a reduction in injury and violent encounters. This is consistent with the literature [16] since the highest cause of death in children and adolescents in Brazil is due to injury and firearms. Thus, during the pandemic lockdown, adolescents had restricted movement, which could have led to a decline in the number of admissions due to reduced violent encounters.

Number of admissions and LOS (days)

Regarding the number of admissions for surgical procedures conducted, thoracic surgery indicated no statistically significant difference across all periods (Table 2). Thoracic surgeries often address critical medical conditions such as lung cancer, esophageal disorders, and other serious respiratory issues. These surgeries are essential and cannot be postponed, even during a pandemic, and thus, hospitals may have continued performing thoracic surgeries with minimal disruption. The decision to proceed with surgeries necessitates a comprehensive patient assessment, which determines whether or not to proceed with surgery [17]. These decisions are mostly made while the patient is admitted. Moreover, thoracic surgery cases tend to be urgent/emergency, requiring immediate medical intervention. The pandemic's influence on urgent thoracic surgery cases may have been negligible, thereby contributing to a steady trend.

The LOS for thoracic surgery decreased (5.1%) between the pre-pandemic and pandemic periods and revealed no statistically significant variation across the entire study period (Table 3). This finding aligns with the stability observed in the number of admissions, prompting us to assume a similar stable trend in the LOS. Although hospitals sought to reduce patient exposure, complex surgical procedures such as thoracic surgery tend to be associated with longer LOS since they carry substantial implications that hinder early discharge. The LOS for all other surgeries had a statistically significant difference (decrease) in LOS. Endocrine gland surgery, surgery of the visual apparatus, and breast surgery had the highest decrease in LOS, at 19.7%, 16.2%, and 16.1%, respectively. This observation corroborates with and confirms a directly proportional relationship with the number of admissions. Our findings align with previous studies [18,19], which reported reductions in LOS for surgical patients during the pandemic and attributed it to effective management strategies and the swift discharge of non-COVID-19 patients employed during the pandemic. However, the study by Rocco et al. [20] reported a statistically significant increase in the average LOS during the pandemic. Other factors reported to shorten the LOS during the pandemic include increased demand for hospital beds and non-COVID-19 patients refraining from hospitals to avoid COVID-19 [21].

Furthermore, surgical guidelines recommended the deferral of non-urgent surgical procedure utilization as a safe public health strategy in the setting of infectious outbreaks [14], for example, breast reconstruction surgery, due to the potential risk of contracting COVID-19 [22]. This is in agreement with the findings of this study, indicating a decreased number of admissions and LOS for non-urgent surgeries in the same period, especially for endocrine surgery, which recorded the highest decrease (19.9%), followed by oral and maxillofacial surgery (17.3%). Given the elevated risk of COVID-19 infection in immunocompromised patients, for example, those undergoing oncological surgery, guidelines recommended delaying surgeries if feasible to minimize intrahospital COVID-19 transmission and making decisions based on the pandemic's impact on the health facility [23]. It is worth noting that surgeons performing more invasive procedures, such as ear, nose, and throat, thoracic, foregut, and endoscopy, were at higher risk of contracting COVID-19 during the pandemic due to the aerosol transmission of the virus [14].

Our study found a statistically significant difference in the number of admissions for surgery in oncology across the study period, a 4.5% decrease, which is close to the 5.6% reduction reported by the study of Luizeti et al. [23], inferring that Brazil showed a tendency to opt for surgical intervention for most oncology cases. This is because the management of oncological cases is crucial for health systems as treatment delays have severe consequences for patients and society. Literature indicates that postponing malignancy treatment by over one month leads to worse prognoses for rectal and breast cancer in high-volume centers and increases mortality rates [24].

Contrary to the study of Luizeti et al. [23], despite Brazil being one of the countries with the highest cesarean rates and guidelines advocating that the type of delivery should not be influenced by maternal COVID-19 infection, our findings demonstrated a statistically significant difference for obstetric surgery. Surgery of the visual apparatus; surgery of the digestive system, associated organs, and abdominal wall; surgery of the genitourinary system; and oral and maxillofacial surgery exhibited higher admission rates during the post-pandemic period compared to baseline. Oral and maxillofacial surgery also showed the same trend for LOS (Table 3). This could be attributed to the backlog of elective surgical cases that had been deferred since the start of 2020, and it is expected that with time, values will approach baseline.

Elective surgeries had a pronounced decline in the number of admissions and LOS (13% and 9.3%, respectively) between 2019 and 2020 compared to urgent surgeries, which experienced a slight decrease in admissions and LOS (2.4% and 2.8%, respectively) in the same period (Table 4). This decline in elective surgeries is in line with the finding by Bigoni et al. [10] that surgeries of low and medium complexity, transplants, and screening procedures were the most affected by the reduction of more than 50% throughout 2020. Worldwide, most health facilities have canceled or postponed non-urgent procedures to prevent overload and enable reorganization of the healthcare systems [25]. The Brazilian health system also implemented the cancellation of non-urgent surgical procedures under the recommendation of the Brazilian National Health Surveillance Agency [23], aligning with international surgical institutions [22], and this corroborates our findings. The number of admissions for elective surgery returned to baseline in the post-pandemic period, highlighting the reopening of health facilities and surgical services to routine operations and tackling of the accumulated backlog from postponed surgeries.

The LOS for elective or urgent surgeries did not return to baseline (Table 4), probably because a lengthy hospital stay during the pandemic would place the patient at risk of COVID-19 infection. It can also be hypothesized that short LOS are becoming the norm as a consequence of the protocols that were established during the pandemic. Urgent surgeries did not return to baseline and were not largely affected by the pandemic since they were performed throughout the period and thus did not have a backlog but rather a decline perhaps due to surgical service provision. However, some authors [26-28] have reported results that differ from this study. The study by Wang et al. [27] reported a notable surge in hospital admissions for emergency surgery (20%-30%), which was ascribed to a rise in the volume of patients seeking hospital care due to critical, life-threatening ailments. It is worth acknowledging that the LOS can be impacted by various variables, encompassing the complexity of procedures, the age and well-being of patients, and the accessibility of resources.

This study presents certain limitations. The data was obtained solely from one country, which might limit the generalizability of the results to other nations. Despite these constraints, this study yields valuable insights into the effect of the pandemic on surgical procedures.

## Conclusions

The analysis of population characteristics, including age, sex, and region, revealed a noticeable decrease in hospital admissions during the pandemic, followed by a subsequent rebound toward pre-pandemic levels during the recovery and post-pandemic phases. Furthermore, there was a decline in both the number of admissions and the length of hospital stay for various surgical procedures during the pandemic, with a gradual return to baseline levels (pre-pandemic) in the recovery and post-pandemic periods. Interestingly, no statistically significant differences were observed for thoracic surgery across all periods, reflecting the emergency/urgency of thoracic surgeries compared to other studied surgeries. Hence, we conclude that the pandemic's influence on thoracic surgery cases may have been negligible, thereby contributing to a steady trend.
